# Measurements of the Electrical Conductivity of Monolayer Graphene Flakes Using Conductive Atomic Force Microscopy

**DOI:** 10.3390/nano11102575

**Published:** 2021-09-30

**Authors:** Soomook Lim, Hyunsoo Park, Go Yamamoto, Changgu Lee, Ji Won Suk

**Affiliations:** 1 School of Mechanical Engineering, Sungkyunkwan University, Suwon 16419, Gyeonggi-do, Korea; growing18@naver.com (S.L.); park102811@gmail.com (H.P.); peterlee@skku.edu (C.L.); 2 Department of Aerospace Engineering, Tohoku University, 6-6-01 Aramaki-Aza-Aoba, Aoba-ku, Sendai 980-8579, Japan; gyamamoto@tohoku.ac.jp; 3 SKKU Advanced Institute of Nanotechnology (SAINT), Sungkyunkwan University, Suwon 16419, Gyeonggi-do, Korea; 4 Department of Smart Fab. Technology, Sungkyunkwan University, Suwon 16419, Gyeonggi-do, Korea

**Keywords:** graphene, reduced graphene oxide, flakes, conductive atomic force microscopy, electrical conductivity

## Abstract

The intrinsic electrical conductivity of graphene is one of the key factors affecting the electrical conductance of its assemblies, such as papers, films, powders, and composites. Here, the local electrical conductivity of the individual graphene flakes was investigated using conductive atomic force microscopy (C-AFM). An isolated graphene flake connected to a pre-fabricated electrode was scanned using an electrically biased tip, which generated a current map over the flake area. The current change as a function of the distance between the tip and the electrode was analyzed analytically to estimate the contact resistance as well as the local conductivity of the flake. This method was applied to characterize graphene materials obtained using two representative large-scale synthesis methods. Monolayer graphene flakes synthesized by chemical vapor deposition on copper exhibited an electrical conductivity of 1.46 ± 0.82 × 10^6^ S/m. Reduced graphene oxide (rGO) flakes obtained by thermal annealing of graphene oxide at 300 and 600 °C exhibited electrical conductivities of 2.3 ± 1.0 and 14.6 ± 5.5 S/m, respectively, showing the effect of thermal reduction on the electrical conductivity of rGO flakes. This study demonstrates an alternative method to characterizing the intrinsic electrical conductivity of graphene-based materials, which affords a clear understanding of the local properties of individual graphene flakes.

## 1. Introduction

Since the intriguing electronic properties of graphene have been experimentally observed in mechanically cleaved graphene [1,2,3], it has gained significant attention in various applications owing to its remarkable electrical, mechanical, thermal, and optical properties [4,5,6,7]. Two dominant methods for obtaining monolayer graphene in large quantities or large areas have been developed to overcome the limitations of mechanical cleavage from graphite. Chemical vapor deposition (CVD) on metals produces high-quality monolayer graphene with a nearly unlimited length [8]. In contrast, the exfoliation of oxidized graphite into a monolayer and the subsequent reduction produce large quantities of graphene flakes [9].

Electrical conductivity is one of the most important properties of synthesized graphene. However, the intrinsic electrical conductivity of graphene is significantly affected by its atomic and chemical structures. For example, the presence of grain boundaries in polycrystalline CVD-grown graphene deteriorates its electrical conductivity owing to the scattering at the grain boundary interfaces [10]. Graphene oxide (GO) obtained by exfoliation of graphite oxide contains oxygen functional groups such as hydroxyl, carboxyl, and epoxide [11]. Therefore, GO is electrically insulating and requires an additional chemical or thermal reduction for applications in electrically conductive devices or materials. However, the removal of oxygen functional groups in GO is dependent on the reduction conditions, which significantly affects the electrical conductivity of reduced graphene oxide (rGO) [12]. In addition, even after the removal of the oxygen functional groups, atomic defects exist in rGO; these originate from the strong oxidation–reduction process [13]. In this respect, measurements of the intrinsic electrical properties of monolayer graphene are highly correlated with its atomic and chemical structures and are of considerable importance in gaining an understanding of synthesized graphene.

The electrical properties of monolayer graphene have been investigated using several methods. To characterize individual graphene flakes, micro-sized metal electrodes were patterned on a graphene flake using photolithography techniques. Although this method has been extensively used to study the electrical properties of graphene flakes, such as mechanically exfoliated graphene and individual GO flakes [1,12], it requires complex microfabrication techniques, including positioning graphene flakes, coating a photoresist layer, depositing a metal layer, and removing the photoresist, which hinder the rapid characterization of mass-produced graphene materials. Macroscopic samples obtained by assembling individual graphene flakes have been widely used to characterize the electrical properties of mass-produced graphene-based materials, including papers fabricated by the layer-by-layer assembly of GO flakes and subsequent reduction [14], compressed graphene powders [15], and composites comprising graphene and polymer matrix [16]. However, the macroscopic assembly of graphene materials cannot fully reflect the electrical properties of individual graphene flakes because other factors, such as the morphology of graphene flakes, interactions between graphene and the polymer, and the non-uniform dispersion of graphene, may affect the electrical properties of the macroscopic assembly. Recently, conductive atomic force microscopy (C-AFM) has been utilized to study the local electrical properties of graphene sheets [17,18,19]. However, there is still a demand for characterizing individual graphene flakes synthesized using scalable production methods.

In this study, we employed C-AFM to directly measure the electrical conductivity of individual graphene flakes prepared by two prominent scalable synthesis methods: CVD on copper and the thermal reduction of GO. Without using any additional materials for electrical measurements, a graphene flake connected to a pre-fabricated electrode was scanned using an AFM tip, which generated an in-plane current distribution. The current change was analyzed as a function of the distance from the electrode to extract the electrical conductivity of the individual graphene flakes. This method was applied to CVD-grown monolayer graphene flakes. Furthermore, individual rGO flakes were characterized as a function of the degree of thermal reduction.

## 2. Materials and Methods

### 2.1. Preparation of Monolayer Graphene Flakes

High-quality sub-monolayer graphene was synthesized on copper foil using low-pressure chemical vapor deposition (LPCVD) [8]. Copper foil (46986, Alfa Aesar, Haverhill, MA, USA) was immersed in acetic acid to remove the native copper oxide. The cleaned copper foil was placed in a tube furnace and annealed with hydrogen at 950 °C for 60 min prior to graphene growth. A mixture of methane and hydrogen was introduced to synthesize monolayer graphene on copper at 950 °C. The growth time was adjusted to synthesize flower-shaped sub-monolayer graphene; the injection of methane was stopped before the individual graphene flakes merged to form a continuous layer.

The polymer-assisted wet transfer method was used to place CVD-grown monolayer graphene flakes on SiO_2_/Si [20,21]. Poly(methyl methacrylate) (PMMA, Mw 996,000, Sigma-Aldrich, St. Louis, MO, USA) dissolved in chlorobenzene (15 mg/mL, Sigma-Aldrich, St. Louis, MO, USA) was spin-coated onto the graphene/copper foil. After drying in air, the copper foil was etched by placing the sample on the surface of an ammonium persulfate solution (0.1 M, Sigma-Aldrich, St. Louis, MO, USA). The PMMA/graphene film was moved to the surface of water several times to rinse off the residue of the etchant. The PMMA/graphene film was moved to a SiO_2_/Si substrate, and the PMMA was removed with acetone after drying completely.

To obtain individual rGO flakes, GO flakes dispersed in water (GO-A-400, Grapheneall, Siheung, Korea) were spread on a SiO_2_/Si substrate after centrifuging the solution at 3000 rpm for 1 h and taking out the upper part of the solution. The GO flakes on SiO_2_ were converted to rGO flakes via thermal annealing with hydrogen (5 sccm) and argon (80 sccm) at atmospheric pressure for 1 h. The samples were annealed at two different temperatures (300 and 600 °C) to change the reduction degree of the rGO flakes.

### 2.2. Electrical Measurements Using C-AFM

The electrical conductivities of the individual graphene flakes were characterized using C-AFM (E-sweep/Nanonavi station, Hitachi High-Tech Science Co., Tokyo, Japan). Metal electrodes (Cr (10 nm)/Au (40 nm)) were pre-fabricated on a SiO_2_/Si substrate (285 nm thick SiO_2_ and highly doped Si with a resistivity of 0.001–0.005 Ω∙cm) using photolithography and metal deposition. After placing graphene flakes on the SiO_2_/Si substrate, the electrical conductance of a graphene flake connected to the electrode was measured using contact-mode C-AFM with a metal-coated tip (Si tip coated with Pt/Ir, SCM-PIT-V2, Bruker, Billerica, MA, USA) with an elastic constant of 3 N/m and a resonance frequency of 75 kHz. The electrical current was measured as a function of the applied bias voltage. All the measurements were performed at room temperature in the air.

**Figure 1:**
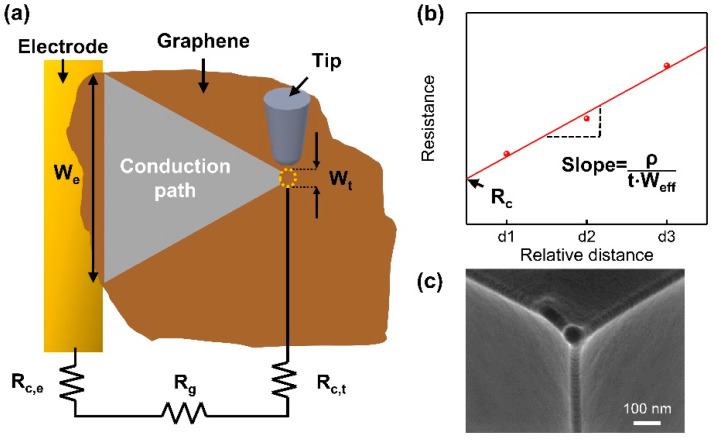
(**a**) Schematic illustration of the electrical measurements of graphene flakes using C-AFM. (**b**) Schematic illustration of the resistance profile as a function of the distance between the tip and the electrode. (**c**) SEM image of the AFM tip.

Figure 1a shows the electrical measurements of the graphene flakes using C-AFM. A graphene flake placed on a SiO_2_/Si substrate was in contact with a pre-fabricated electrode. An AFM tip coated with metal made physical contact with a graphene flake, creating a closed circuit. A constant voltage was applied to the electrode, and the current flowing through the graphene flake was measured between the AFM tip and the electrode. By scanning the tip over the graphene flake, the resistance profile converted from the measured current was obtained as a function of the distance (L) between the tip and the electrode. The total resistance (R_total_) is the sum of the resistance of graphene (R_g_) and the contact resistance (R_c_). Because the C-AFM-based electrical measurement consists of graphene, an AFM tip, and an electrode, the total contact resistance (R_c_) includes the contact resistances of the tip (R_c,t_) and the electrode (R_c,e_) with graphene (Figure 1a).

(1) $$R_{total}=R_{g}+R_{c,e}+R_{c,t}=R_{g}+R_{c}$$

By considering the triangular region formed by the tip and the electrode side as the electrical conduction path, it is possible to estimate the resistance of graphene (R_g_) using the following equation [19,22]:

(2) $$R_{g}=\rho \frac{L}{tW_{eff}}=\rho \frac{L}{t}\frac{\ln\left(\frac{W_{e}}{W_{t}}\right)}{\left(W_{e}-W_{t}\right)}$$

(3) $$W_{eff}=\frac{W_{e}-W_{t}\;}{\ln\left(\frac{W_{e}}{W_{t}}\right)}$$

where ρ is the resistivity of graphene, t is the thickness of graphene, W_e_ is the connected length between the graphene and the electrode, W_t_ is the diameter of the contact area under the tip, and W_eff_ is the effective conduction width of the graphene flakes. After obtaining the resistance profile as a function of the distance of the tip from the electrode, the contact resistance is estimated from the intercept at the *y*-axis (Figure 1b), which is similar to the transmission line measurement (TLM) method [23]. The slope of the linearly fitted curve provides the resistivity (the reciprocal of the conductivity) of the graphene flakes using the following equation (Figure 1b):

(4) $$\mathrm{slope}=\frac{\rho }{tW_{eff}}$$

Because it is difficult to measure the actual contact area of the AFM tip, the Hertz contact model was employed to estimate the diameter of the tip contact area (W_t_) [19]. Based on the Hertz contact model, the applied force is related to the radius of the AFM tip (R_tip_), elastic indentation depth (δ), and reduced Young’s modulus (K) as follows [24]:

(5) $$F=KR_{tip}{}^{1/2}\delta^{3/2}$$

The reduced Young’s modulus (K) is defined as follows:

(6) $$\frac{1}{K}=\frac{3}{4}\left(\frac{1-\nu_{s}^{2}}{E_{s}}+\frac{1-\nu_{t}^{2}}{E_{t}}\right)$$

where E_t_ and E_s_ are the elastic moduli of the tip and the sample, respectively. ν_t_ and ν_s_ are the Poisson’s ratios of the tip and the sample, respectively. The radius of the tip was measured to be 33 nm from the SEM image, as shown in the inset of Figure 1c.

However, the Hertz model only considers the contact pressure inside the contact area in the absence of adhesive interactions [25]. Thus, the Derjaguin–Műller–Toporov (DMT) contact model was used to estimate the contact area because a previous study had shown that adhesive interactions between a sharp indentation tip and monolayer graphene were in the DMT regime [26,27]. Based on the DMT contact model, the adhesion force between the tip and the sample (F_ad_) is involved in the estimation of the contact radius (a) as follows [24]:

(7) $$a={\left[\frac{R_{tip}}{K}\left(F+F_{ad}\right)\right]}^{1/3}$$

The adhesion force (F_ad_) was estimated according to the force–distance curve from the AFM indentation [24,28]. The elastic moduli of CVD-grown graphene and rGO flakes were assumed to be 1 and 0.25 TPa, respectively, according to previous reports [5,29]. 

### 2.3. Characterization of Graphene Materials

The morphology of the monolayer graphene flakes was characterized using scanning electron microscopy (SEM, JSM-7600, Jeol, Tokyo, Japan). The changes in the chemical structures of GO and rGO were investigated using X-ray photoelectron spectroscopy (XPS, ESCALAB-250, Thermo-Scientific, Waltham, MA, USA) with monochromated Al Kα radiation. The C 1s core-level spectra were deconvoluted with Gaussian–Lorentzian functions after a background signal correction using the Shirley profile [30]. The sp^2^-hybridized carbon (C=C) was modeled using the asymmetric Doniach–Sunjic peak shape [31,32]. In addition, Raman spectroscopy (ALPHA300M with a 532 nm excitation laser, WiTec, Ulm, Germany) was used to characterize the graphitic structures of CVD-grown graphene, GO, and rGO.

## 3. Results and Discussion

### 3.1. Electrical Conductivity of CVD-Grown Monolayer Graphene Flakes

**Figure 2:**
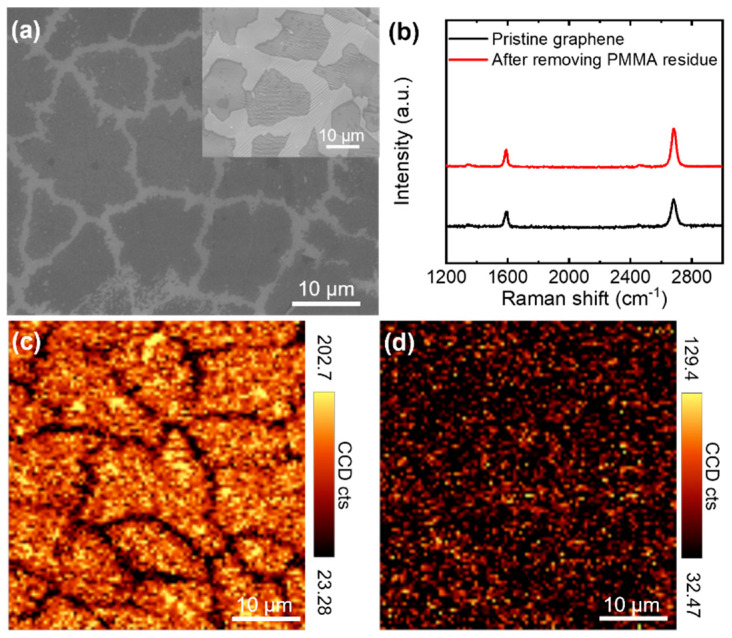
(**a**) SEM image of CVD-grown monolayer graphene flakes transferred onto SiO_2_. The inset shows the SEM image of monolayer graphene grown on the surface of copper foil. (**b**) Raman spectra of CVD-grown monolayer graphene placed on SiO_2_ before and after the removal of the polymer residue. (**c**,**d**) Raman maps for the integrated intensity of (**c**) the G (1542–1642 cm^−1^) and (**d**) D bands (1314–1414 cm^−1^).

The intrinsic electrical conductivity of the CVD-grown graphene was characterized using the C-AFM measurement method. To isolate a graphene flake for electrical measurements, the growth of monolayer graphene was stopped by turning off the methane flow before forming continuous graphene on a copper foil. Therefore, many graphene flakes were formed on the surface of the copper foil (inset of Figure 2a). They were transferred onto SiO_2_/Si for electrical measurements, as shown in Figure 2a. Because the growth of graphene was stopped prior to the formation of the continuous film, the individual graphene flakes did not include grain boundaries [33], implying that the electrical measurements on these graphene flakes could provide the electrical conductivity of single-crystal graphene.

Raman spectra obtained from the graphene flakes show the characteristic features of high-quality monolayer graphene (Figure 2b); the peak intensity of the 2D band at ~2680 cm^−1^ was higher than that of the G band at ~1580 cm^−1^, and there was a minimal D band at ~1350 cm^−1^ [8]. Moreover, the Raman map for the integrated intensity of the G band (1542–1642 cm^−1^) confirms the existence of monolayer graphene flakes over a large area (Figure 2c), while the Raman map for the D band (1314–1414 cm^−1^) indicates a negligible defect distribution in the flakes (Figure 2d). 

**Figure 3:**
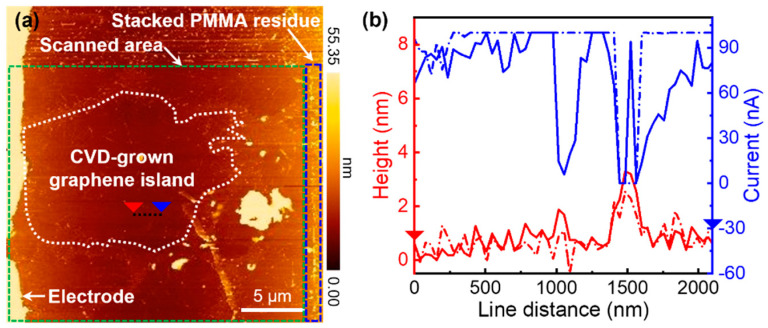
(**a**) AFM topography image of a CVD-grown monolayer graphene flake on SiO_2_ after the removal of the polymer residue. (**b**) The line profile of the current and morphology of the flake along the dashed line marked in (**a**).

Because CVD-grown graphene flakes were covered with a thin polymer layer during the wet transfer process, polymer residues on top of graphene could not be avoided even after removing the polymer with acetone [34]. Because the residual polymer layer might hinder the observation of the intrinsic electrical conductivity of graphene, it was removed through physical sweeping with an AFM tip in a contact-mode scan [35,36]. After scanning, the polymer residue on graphene was removed and stacked on the right side of the scanned area, as shown in Figure 3a. Due to the removal of the rough polymer residue, the root-mean-square (RMS) roughness decreased from 1.45 nm to 1.07 nm, which was in good agreement with a previous study [35]. Therefore, the local current increased after the removal of the polymer residue (Figure 3b). In addition, contact-mode scanning was performed at a slow rate to avoid defect generation. The unchanged D band in the Raman spectra after scanning indicated that the polymer removal process did not affect the quality of graphene (Figure 2b,d). 

**Figure 4:**
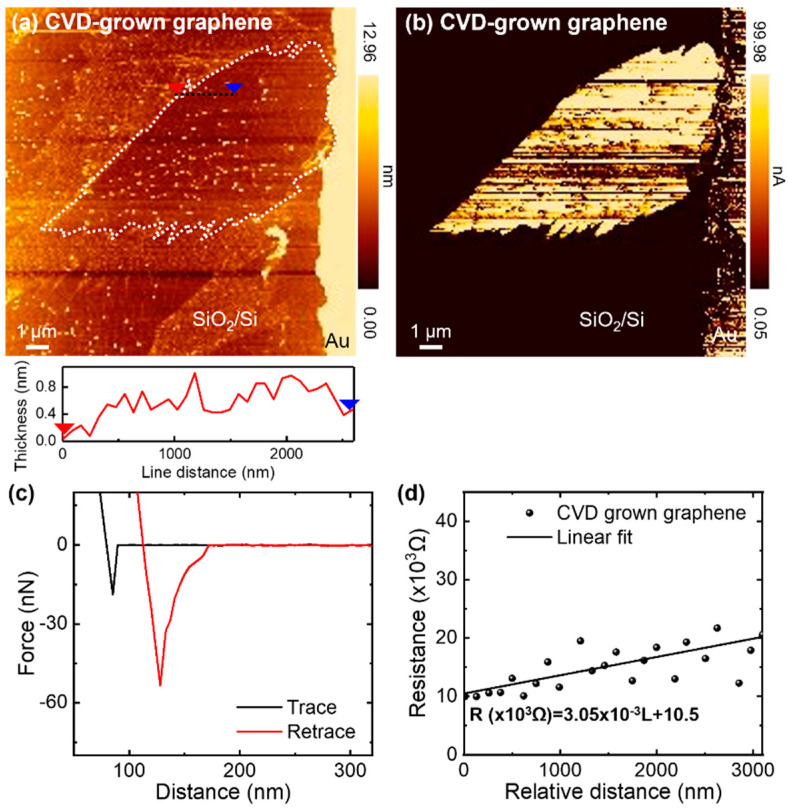
(**a**,**b**) AFM images for the (**a**) topography and (**b**) current distribution of the CVD-grown monolayer graphene flake on SiO_2_. (**c**) Force–distance curve. (**d**) Resistance profile of the graphene flake as a function of the distance from the tip to the electrode.

Figure 4a,b shows the AFM topography and current map of a CVD-grown graphene flake, respectively. The length of graphene connected to the electrode (W_e_) was 4.33 μm. The force–distance curve exhibited a pull-off force of 57.6 ± 13 nN when 3 nN was applied to the cantilever (Figure 4c). Therefore, the contact area estimated using the DMT contact model was 13.487 nm^2^ (W_t_ = 4.144 nm). Figure 4d shows the measured resistance as a function of the distance from the AFM tip to the electrode. By fitting the resistance curve with the exception of the low conductive spots, the electrical conductivity of CVD-grown monolayer graphene flakes was approximately estimated to be 1.46 ± 0.82 × 10^6^ S/m. This is close to the sheet resistances of CVD-grown monolayer graphene measured by the van der Pauw method in previous studies [21,37]. 

### 3.2. Electrical Conductivity of Thermally Reduced Graphene Oxide Flakes

The electrical conductivity of rGO flakes obtained by thermally reducing the GO flakes placed on SiO_2_/Si was characterized by C-AFM measurements. GO flakes were thermally annealed at two different temperatures (300 and 600 °C) to investigate the effect of the reducing temperature on the electrical conductivity of the rGO flakes.

**Figure 5:**
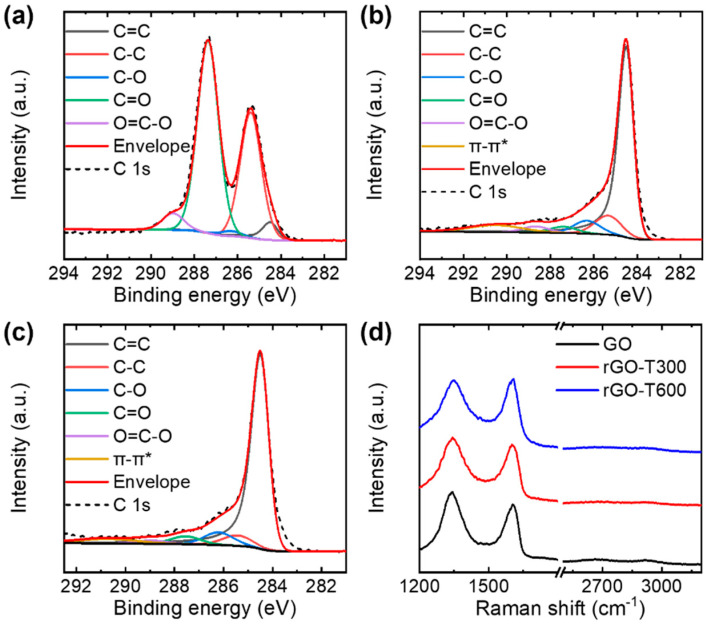
(**a**–**c**) XPS C 1s spectra of (**a**) GO and (**b**,**c**) rGO flakes. Thermal annealing was performed at (**b**) 300 and (**c**) 600 °C. (**d**) Raman spectra of GO and rGO flakes. rGO-T300 and rGO-T600 denote rGO flakes annealed at 300 and 600 °C, respectively.

**Figure 6:**
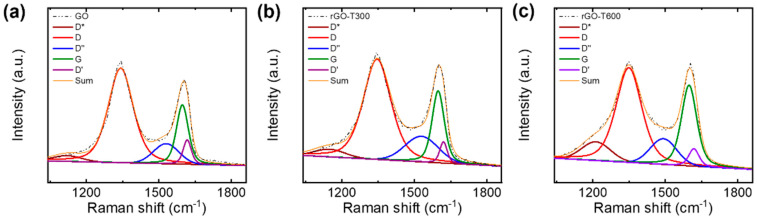
Deconvolution of Raman spectra of (**a**) GO and (**b**,**c**) rGO flakes.

The GO and rGO flakes were characterized using XPS and Raman spectroscopy. The XPS C 1s core-level spectra were deconvoluted with sp^2^-hybridized carbon (C=C) at 284.5 eV, sp^3^-hybridized carbon (C-C) at 285.3 eV, C–O at 286.3 eV, C=O at 287.6 eV, and O=C–O at 288.8 eV (Figure 5a–c) [38,39]. In addition, the XPS spectra of rGO flakes included π–π* transitions at 290.7 eV [40,41,42]. Based on the XPS analysis, the GO flakes had a C/O ratio of 1.5, which is similar to that of typical GO [43]. The thermal treatment partially removed oxygen functional groups, thereby increasing the C/O ratio to 4.6 and 5.3 for the rGO flakes annealed at 300 and 600 °C, respectively. The Raman spectra of GO and rGO showed typical D and G bands at ~1350 and ~1580 cm^−1^, respectively (Figure 5d) [44]. To obtain an accurate estimation of the intensity ratio of the D peak to the G peak, I_D_/I_G_, the broad D and G bands were deconvoluted into five components, as shown in Figure 6: G (~1580 cm^−1^), D (~1350 cm^−1^), D* (~1150–1200 cm^−1^), D″ (~1500–1550 cm^−1^), and D′ (~1620 cm^−1^) [45,46,47,48]. Three pseudo-Voigt (for D, G, and D’ peaks) and two Gaussian (for D* and D″ peaks) functions were used to fit the Raman spectra in the range of 1000–1800 cm^−1^ [48]. The analysis of the Raman spectra revealed that I_D_/I_G_ decreased from 1.56 ± 0.06 for the GO flakes to 1.32 ± 0.07 and 1.08 ± 0.06 for the rGO flakes annealed at 300 and 600 °C, respectively. The change in the characteristics of the Raman spectra indicates the removal of oxygen functional groups [49,50], which is in good agreement with the XPS analysis.

**Figure 7:**
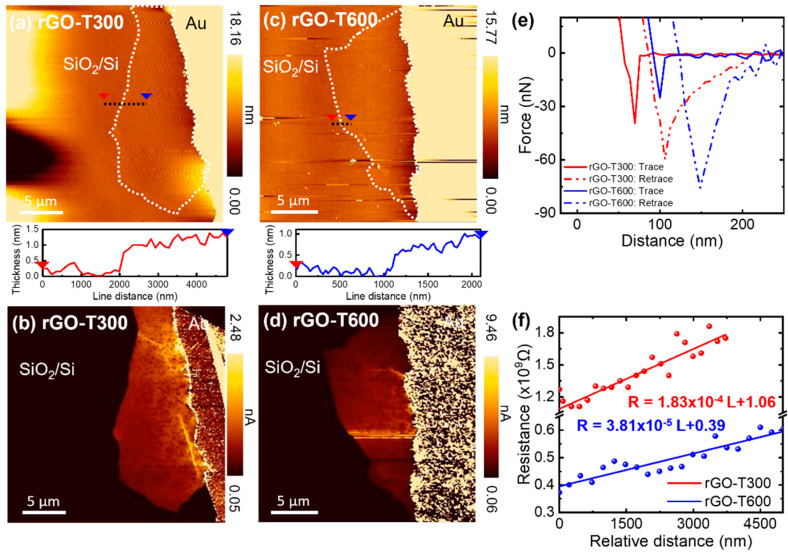
(**a**,**b**) AFM images for the (**a**) topography and (**b**) current distribution of the rGO flake annealed at 300 °C. (**c**,**d**) AFM images for the (**c**) topography and (**d**) current distribution of the rGO flake annealed at 600 °C. (**e**) Force–distance curves. (**f**) Resistance profiles of the rGO flakes as a function of the distance from the tip to the electrode.

Figure 7a,c shows the AFM topography of the rGO flakes synthesized by the thermal annealing of GO flakes at 300 and 600 °C, respectively. The thickness of the rGO annealed at 300 °C was ~1.0 nm, whereas the rGO flake annealed at 600 °C had a thickness of ~0.6 nm, confirming the greater removal of oxygen functional groups at a higher temperature. Figure 7b,d presents the corresponding current maps of the rGO flakes. The force–distance curves of the rGO flakes annealed at 300 and 600 °C provided pull-off forces of 63.0 ± 10.4 nN and 74.5 ± 5.5 nN, respectively (Figure 7e). This shows that the effective contact areas between the tip and the rGO flake were 18.643 nm^2^ (W_t_ = 4.872 nm) and 20.750 nm^2^ (W_t_ = 5.140 nm) at annealing temperatures of 300 and 600 °C, respectively. Therefore, using the given equation to fit the resistance–distance curves, the electrical conductivities were estimated to be 2.3 ± 1.0 and 14.6 ± 5.5 S/m for 300 and 600 °C annealing samples, respectively (Figure 7f). This indicates that a greater reduction of GO provided a higher electrical conductivity along with a higher C/O ratio and lower I_D_/I_G_. Several previous studies have investigated the electrical conductivity of isolated graphene flakes. Monolayer rGO flakes with a C/O ratio of ~4 synthesized by the photoreduction of GO using ultraviolet light irradiation in vacuum exhibited an electrical conductivity of 0.20 S/m, which was directly measured by C-AFM [51]. Moreover, four-probe measurements using micropatterned electrodes deposited on GO flakes showed electrical conductivities ranging from 33 to 85 S/m after a partial reduction by thermal annealing in vacuum [43]. A similar measurement method showed that monolayer rGO flakes obtained by the chemical reduction of GO via hydrazine had electrical conductivities ranging between 5 and 200 S/m [52]. Considering that GO flakes were partially reduced by thermal annealing at relatively low temperatures, the electrical conductivities reported in previous studies are comparable to those measured by C-AFM in this study.

## 4. Conclusions

The electrical conductivities of isolated graphene flakes were investigated using C-AFM and an analytical analysis. Monolayer graphene flakes obtained by CVD on copper foil and the thermal reduction of GO were characterized using C-AFM. High-quality CVD-grown monolayer graphene flakes transferred onto SiO_2_/Si exhibited a high electrical conductivity comparable to that measured by the van der Pauw method. In addition, GO flakes placed on SiO_2_/Si were thermally reduced at two different temperatures. Annealing the rGO flakes at higher temperatures resulted in a higher electrical conductivity, which was confirmed by an increase in the C/O ratio and a decrease in I_D_/I_G_. This study demonstrates that the C-AFM method is capable of estimating the local electrical conductivity of individual graphene flakes. In addition, this study provides a better understanding of graphene materials synthesized using the two major scalable production methods.

## Data Availability

Not applicable.
